# The Influence of Technical and Contextual Variables of the Last Stroke on Point Outcome in Men’s and Women’s Singles Badminton

**DOI:** 10.3389/fpsyg.2021.802179

**Published:** 2022-01-10

**Authors:** Yi Sheng, Qing Yi, Miguel-Ángel Gómez-Ruano, Peijie Chen

**Affiliations:** ^1^School of Physical Education and Sport Training, Shanghai University of Sport, Shanghai, China; ^2^Shanghai Key Laboratory of Human Performance, Shanghai University of Sport, Shanghai, China; ^3^Facultad de Ciencias de la Actividad Física y del Deporte (INEF), Universidad Politécnica de Madrid, Madrid, Spain; ^4^School of Kinesiology, Shanghai University of Sport, Shanghai, China

**Keywords:** badminton, racket, match analysis, notational analysis, scoring pattern

## Abstract

The purpose of this study was to identify the effects of the technical and context-related variables of last strokes in rallies on the point outcomes of both men’s and women’s players in elite singles badminton matches. A total of 100 matches during the 2018 and 2019 seasons were analyzed, and the data of 4,080 men’s rallies and 4,339 women’s rallies were collected. The technical variables including strokes per rally, forehand strokes, overhead strokes, and defensive action, and the context-related variables including game status, result against serve, importance of rally, and importance of set, were modeled with Probit regression modeling as the predictor variables. The binary variables of “winner or not” and “error or not” were considered the response variables. The results showed that defensive actions had the greatest impacts on the winners and errors of both the men’s and women’s singles players, and the forehand and overhead strokes were negatively associated with the winners and errors of the women’s singles players and the winners of the men’s singles players. No significant effects were found for the strokes per rally on the winners and errors of the men’s singles players, while significant effects were found for the women’s singles players. The context-related variables appeared to have positive effects on the winners and negative effects on the errors of both sexes. These findings can provide important insights for coaches and players to evaluate their performances of last strokes in rallies and to improve training interventions and match tactics and strategies.

## Introduction

Badminton matches are characterized by high intensity and intermittent efforts involving numerous repetitive actions and consecutive movements during rallies (Manrique and Gonzalez-Badillo, 2003; Abdullahi and Coetzee, 2017). Technical, physical, and temporal variables have been widely used in previous studies to interpret and quantify the match characteristics of badminton players, and the technical aspect has gained the most interest of the academic community, especially the singles modality (Phomsoupha and Laffaye, 2015; Chiminazzo et al., 2018; Gómez-Ruano et al., 2020a). Notational methodologies have previously been used to depict and assess the technical characteristics (serving patterns, stroke effectiveness, the effects of context-related variables, and the dynamics of scoring performance) (Phomsoupha and Laffaye, 2015; Barreira et al., 2016; Chiminazzo et al., 2018; Gómez-Ruano et al., 2020a). However, most of the studies were focused on observational analysis by comparing the technical variables of different groups (Abian-Vicen et al., 2013; Torres-Luque et al., 2019; Pérez-Turpin et al., 2020), which provided limited realistic information for coaches and players to prepare for competitions and training sessions. To date, little explorative analysis has been conducted that attempts to investigate the influencing factors of players’ scoring performance (Gómez-Ruano et al., 2021).

Due to technological advances, the improved match performance tracking system can provide multidimensional and detailed match performance data for performance analysts to conduct more elaborate analyses, allowing them to explore the keys to succeeding in a match (Hughes et al., 2007; Yi et al., 2020b). Given the focus on the investigation of key factors for the match outcome in badminton, it is of great importance to evaluate the players’ performances of the last stroke in the rallies. The authors of the available literature have paid more attention to collecting and analyzing the frequency data of different stroke types based on the whole match scale (clear, smash, lob, cut, drive, etc.) (Chiminazzo et al., 2018), while only a few studies have examined the distribution of last strokes per rally, identifying the unforced error, smash, net, and drop as the most decisive actions to end rallies (Abian-Vicen et al., 2013; Abián et al., 2014). However, investigations into the influencing factors of point outcome (winner, unforced error, and forced error) are still scarce. Evolutionary trends of match characteristics have been previously reported; rally times, the frequency of strokes per second, and the number of shots per rally increase in singles matches over time (Abián et al., 2014; Laffaye et al., 2015). In light of the changes in the playing structure, it is necessary to analyze the relationships between the performance of the last strokes and the point outcome in modern badminton matches, which may provide important information for understanding the scoring patterns.

The significant impact of match context-related variables on the players’ match behaviors has been highlighted in the literature (Gómez-Ruano et al., 2013; Yi et al., 2019), and this research topic has been discussed in depth in other sports, including football, basketball, handball, and more (Liu et al., 2015; Prieto et al., 2016; Zhang et al., 2019; Yi et al., 2020a). However, it has only been very recently that the effects of context-related variables on badminton players have been emphasized by researchers (Gómez-Ruano et al., 2020a,^2021^). It is obvious that players’ performances during rallies may vary dramatically in dynamic situations, for example, the score line, the set number, game points, and the right of service, and these specific situations may place great physiological, psychological, and cognitive loads on players (Ghosh, 2008; Alcock and Cable, 2009; Alder et al., 2019). Players need to adopt different effective technical and tactical actions that make it possible to defeat their opponents during rallies (Hastie et al., 2009; Seth, 2016). Therefore, the effects of context-related variables should not be ignored when analyzing the performance of strokes that players use per rally during the ending rallies. Moreover, the differences between the sexes regarding the temporal, physiological, and notational characteristics have been well-explored in the literature (Cabello et al., 2004; Abian-Vicen et al., 2013; Valldecabres et al., 2017). Abian-Vicen et al. (2013) reported that the smash and drive were used more frequently by men’s players and the drop was used more frequently by women’s players in singles badminton matches. Presumably, the differences may also exist in the influencing factors of point outcome between both sides.

Therefore, in the current study, we aimed to quantify the influences of the technical variables of the last strokes in each rally and the context-related variables on the point outcome for both men’s and women’s players in elite singles badminton matches. It was hypothesized that the players’ performances during the last strokes are affected by stroke techniques and the match situations in men’s and women’s singles badminton.

## Materials and Methods

### Sample

The sample was composed of 100 matches for men’s and women’s singles players (*n* = 50 matches, *n* = 4,080 rallies and *n* = 50 matches, *n* = 4,339 rallies, respectively), which were randomly selected from matches played in professional badminton competitions from 2018 to 2019 (2018 Japan Open, 2018 China Open, 2018 Uber Cup, 2019 Sudirman Cup, 2018 Denmark Open, 2019 All England Open, 2019 Singapore Open, 2019 Malaysia Open, 2019 China Open, 2019 Indonesia Open, 2019 Asia Championships, and 2019 BWF World Championships). All the matches were publicly available on TV^1^ and included all the rallies played within each full match. The data were collected based on videos, and the collecting process was a non-invasive method; only the variables that related to the performance of the last stroke per rally were included in the sample. The ethics committee approval was not required by the university when analyzing the videos available in the public domain.

### Procedure

The observational methodology was non-participant and direct with an observational design: nomothetic, multidimensional and follow-up of multiple players (Anguera et al., 2011). The badminton match actions and events and the match contexts of last stroke per rally were considered as independent observations, and were coded from the footage by four well-trained operators with more than 2 years of experience in performance analysis, using a computerized tracking system jointly developed by Danzle Co., Ltd., and Shanghai University of Sport. The inter- and intra-observer reliabilities of the collected data were tested with good to very good levels (Kappa ≥ 0.83; correlation coefficient *r* ≥ 0.86, intra-class correlation coefficient ≥ 0.87; and typical error of measurement ≤ 0.48) (Altman, 1990; Hopkins, 2000). The point outcome of each rally for each player was identified as one of three types—winner, forced error, and unforced error according to previous studies of single badminton (Laffaye et al., 2015; Gómez-Ruano et al., 2017). The selected technical and context-related variables were defined before the data collection (Table 1). The technical variables included the strokes per rally, the stroking position of the last stroke per rally, and the stroking type of the last stroke per rally. All the stroking types (smash, clear, drive, drop, net, and lob) were defined and classified into two groups, offensive actions and defensive actions (active intention to score a point and no intention to score a point). The classification of strokes was based on the tactical aim of a stroke performed and the shuttlecock trajectory (Laffaye et al., 2015), as some strokes (e.g., lob and clear) can be considered either defensive actions or defensive actions in different situations. Four context-related variables were selected—game status, result against serve, the importance of the rally, and the importance of the set.

**TABLE 1:** Definitions of selected technical and context-related variables.

Type of variable	Variables	Definitions
Technical variables	Strokes per rally	The number of strokes achieved during a rally.
	Stroking position	The position that the shuttlecock was hit in the last stroke per rally, including forehand stroke, backhand stroke, and overhead stroke.
	Stroking type	Technical skills that were used for hitting the shuttlecock in the last stroke per rally, including smash, clear, net, lob, cut, drive, flick, etc.
Context-related variables	Game status	Winning or not before the rally.
	Result against serve	Points scored against the serve or not. The right of service is considered an advantage for the server; a point scored against a serve was recorded if players scored a point when the opponent was the server.
	Importance of rally	Key rally/non-key set. The game point was considered a key rally.
	Importance of set	Key set or non-key set. The “second set is the key set if the opponent won the first set; the third set is the key set if both sides won a set.

### Statistical Analysis

A data normality test (Shapiro–Wilk) was performed for the continuous variables (*P* < 0.05). Then, Probit regression was used to quantify the effects of technical and context-related variables on the point outcome for both the men’s and women’s singles players. Forced error and unforced error were merged into a new variable, named error, and then the binary variables of “winner or not” and “error or not” were selected as the response variables, respectively, for the modeling. As the predictor variables included both continuous variables and categorical variables, two polytomous variables (the stroking position of the last stroke per rally and the stroking type of the last stroke per rally) were transformed into dummy predictor variables in order to examine the specific effects of different positions and types of strokes on the point outcome. The results of the Probit regression were easier to interpret when the dummy variables were limited to two values (0 or 1), with 0 representing the absence of a qualitative attribute and 1 representing its presence. The number of dummy variables could be defined as K−1 when the number of values of the polytomous variable was assumed to be K (Hardy, 1993). Therefore, the stroking position of the last stroke per rally (forehand, backhand, and overhead strokes) was transformed into two dummy predictor variables (forehand and overhead strokes), and the stroking type of the last stroke per rally was transformed into one dummy predictor variable (defensive action). The removed dummy became the base category that the other categories could be compared with. Ultimately, there were four technical variables—strokes per rally, forehand stroke, overhead stroke, and defensive action, and four context-related variables (binary situation)—game status (winning or not before this rally), result against serve (points scored against serve or not), importance of rally (key rally or not), and importance of set (key set or not)—were observed as the predictor variables for the Probit regression models. After the estimations, the command mfx was used to calculate the marginal effects. The marginal effect indicates the effect of a one-unit change in the predictor variable on the change (%) in the probability of the response variables (winner or not and error or not) (Mullahy, 2017). The statistical analyses were performed using the statistical software STATA/SE 16.0 (StataCorp., United States). The level of significance was set at 0.05.

## Results

**FIGURE 1:**
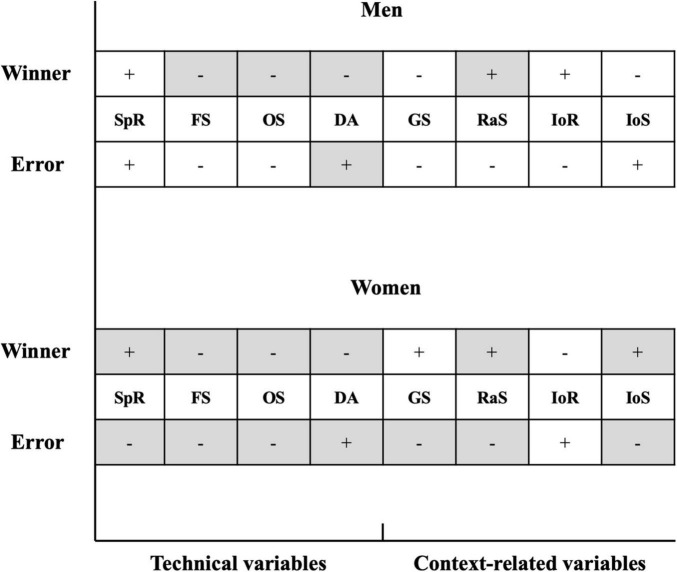
Comparison of identified significant variables between the winner and error for men’s and women’s singles players. + indicates the positive regression coefficient, – indicates the negative regression coefficient. The gray shaded squares indicate the significant relationships between the corresponding variables and the point outcomes. SpR, strokes per rally; FS, forehand stroke; OS, overhead stroke; DA, defensive action; GS, game status; RaS, result against serve; IoR, importance of rally; IoS, importance of set.

The parameter estimates of the Probit regression models are presented in Tables 2, 3, and the results are summarized and visualized in Figure 1. The technical variables of forehand stroke, overhead stroke, and defensive action (marginal effect: ME = −0.58 to −0.10, *P* < 0.05) and the context-related variable of result against serve (ME = 0.085 to 0.176, *P* < 0.05) showed positive and negative relationships, respectively, with the winners of both the men’s and women’s singles players. In addition, positive effects of the number of strokes per rally and the importance of set on the winners were also identified for women’s singles players (ME = 0.085 to 0.176, *P* < 0.05). Only the defensive action (ME = 1.327, *P* < 0.01) from men’s singles players showed a positive relationship with the errors, while all the technical and context-related variables (ME = −0.151 to 1.001, *P* < 0.05) showed significant effects on the errors of women’s singles players, with the exception of the importance of rally.

**TABLE 2:** Parameter estimates for the relationships between winners and the technical and context-related variables for men’s and women’s singles players.

Variables	Winner					
	Men (*n* = 4,080)			Women (*n* = 4,339)		
	*Z*-score	Coef.	ME	*Z*-score	Coef.	ME
Technical variables						
Strokes per rally	1.610	0.004	0.002	6.630	0.020**	0.008
Forehand stroke	–2.030	−0.100*	–0.040	–3.750	−0.176**	–0.070
Overhead stroke	–3.390	−0.210**	–0.084	–5.210	−0.305**	–0.121
Defensive action	–12.870	−0.580**	–0.228	–7.820	−0.322**	–0.128
Situational variables						
Game status	–0.290	–0.012	–0.005	1.390	0.056	0.022
Result against serve	2.050	0.085*	0.034	4.420	0.176**	0.070
Importance of rally	1.740	0.164	0.064	–0.230	–0.020	0.042
Importance of set	–0.960	–0.054	–0.021	2.000	0.107*	0.042
Constant	5.910	0.380**		1.080	0.068	
Pseudo R-squared	0.033			0.024		

*P < 0.05, **P < 0.01.

*Coef., Probit regression coefficient; ME, marginal effect.*

**TABLE 3:** Parameter estimates for the relationships between the errors and the technical and context-related variables for men’s and women’s singles players.

Variables	Error					
	Men (*n* = 4,080)			Women (*n* = 4,339)		
	*Z*-score	Coef.	ME	*Z*-score	Coef.	ME
Technical variables						
Strokes per rally	0.600	0.002	0.001	–3.170	−0.010**	–0.004
Forehand stroke	–0.860	–0.047	–0.018	–2.750	−0.141**	–0.052
Overhead stroke	–0.480	–0.031	–0.012	–2.400	−0.148*	–0.056
Defensive action	26.510	1.327**	0.462	22.370	1.001**	0.351
Situational variables						
Game status	–0.760	–0.034	–0.013	–2.770	−0.118**	–0.044
Result against serve	–0.890	–0.039	–0.015	–3.560	−0.151**	–0.056
Importance of rally	–0.880	–0.088	–0.034	0.870	0.080	0.029
Importance of set	1.260	0.076	0.029	–2.440	−0.137*	–0.052
Constant	–3.430	−0.237**		4.180	0.280**	
Pseudo R-squared	0.172			0.117		

*P < 0.05, **P < 0.01.

*Coef., Probit regression coefficient; ME, marginal effect.*

The number of variables that showed significant relationships with the winners of women’s singles players was higher than the number of variables that showed significant relationships with the errors of men’s singles players, and the effects of the selected variables on the winners and errors of women’s players were very similar; the only difference was in the context-related variable of game status. The defensive actions showed negative relationships with the winners of both the men’s and women’s singles players (ME = −0.58 to −0.322, *P* < 0.01), and also showed positive relationships with the errors of both the men’s and women’s singles players (ME = 1.001 to 1.327, *P* < 0.01). No significant relationships were found for the importance of rally with the winners and errors for both the men’s and women’s singles players. The significant effects of the number of strokes per rally (ME = −0.01 to 0.02, *P* < 0.01) and the importance of set (ME = −0.137 to 0.107, *P* < 0.05) on the winners and errors were only found for women’s singles players.

## Discussion

In the current study, we aimed to model the relationships between the technical and context-related variables of the last stroke per rally and the point outcome (winner and error) for the elite men’s and women’s singles badminton players. The current findings revealed the significant effects of technical and context-related variables on the performance of both men’s and women’s singles players during the last strokes, and it was found that the women’s singles players were more susceptible to the technical and context-related factors than the men’s singles players.

For the men’s singles players, stroking position, stroking type, and result against serve had significant effects on the point outcome of the winner. The positive relationship between the result against serve and the winners indicated that the players were more likely to finish the rally using a winner when the opponent was the server. Serving may potentially help the server to gain some spatial and temporal advantage during rallies (Gómez-Ruano et al., 2020a), but players may perform more aggressively as receivers in order to regain the right of serve. It is worth noting that the forehand and overhead strokes were negatively associated with the winners, which means that men’s singles players finishing the rally using forehand and overhead strokes may decrease the probability of performing winners compared to using backhand strokes. These results are contradictory to the previous researcher’s findings that the overhead stroke was the most popular stroke in singles badminton matches, with more than half of the strokes performed using an overhead (Ghosh et al., 2002). It is easy to understand that the probability of obtaining a winner would be decreased when the men’s singles players adopted defensive actions, such as lob, for the last stroke of the rallies rather than offensive actions, such as a smash, Conversely, the last stroke with no intention to score a point may more likely lead to an error, and this notion could be supported by another current finding that only the defensive actions had significant positive associations with the errors. Surprisingly, the number of strokes per rally, stroking positions, and all match contexts had no impact on the errors of the men’s singles players, especially in the match contexts.

For the women’s singles players, the influencing factors of the winners and errors demonstrated very similar trends, in that all the selected technical and context-related variables showed consistent impacts (significant or non-significant) on winners and errors, except for the game status. However, the majority of technical variables displayed negative effects, and the context-related variables displayed positive effects on winners and negative effects on errors. Specifically, the positive relationship between winners and the number of strokes per rally indicated that the longer the rallies, the higher the probability of achieving the winners, which highlights the importance of physical preparation in singles badminton matches. The players with well-prepared physical capacity may be more likely to disrupt the opponents’ balance and achieve the winners during long rallies (Gómez-Ruano et al., 2020b). The positive relationships between the winners and the context-related variables of result against serve and the importance of set demonstrated that the players who scored points without serving were more likely to be linked to the point outcome of the winner, and players had a higher probability of achieving the winners during the key set and during the initial moments of the set (Gómez-Ruano et al., 2021). In addition, similar to the men’s singles players, the forehand strokes, overhead strokes and defensive actions also had negative effects on the winners of the women’s singles players. More attention should be paid to these negative effects when evaluating the stroking performance of both men’s and women’s singles players, and further research is necessary to explore the potential reasons. The highest number of influencing factors were identified for the errors of women’s singles players. The number of strokes per rally, defensive action, result against serve, and the importance of set showed opposite effects on the errors when compared to their effects on the winners, and the effects of the forehand and overhead strokes on the winners and errors were consistent. The significant effect of the game status was only observed for the errors of the women’s singles players; the probability of occurring errors was lower when a player was winning during a set. This could be partially explained by the fact that winning may bring a psychological advantage to the players during match play (Gómez-Ruano et al., 2021).

Interestingly, defensive action was the only variable that displayed significant effects on the winners and errors of both the men’s and women’s singles players, and the negative and positive effects were observed for winners and errors, respectively. These findings reflect the fact that defensive actions may decrease the frequency of the occurrence of winners and increase the frequency of the occurrence of errors. Notational analyses have been conducted in previous studies to describe the distribution of stroking types of the last stroke in the rallies for different genders (Abian-Vicen et al., 2013), competitions (Abián et al., 2014), and match stages (Chiminazzo et al., 2018), concluding that the smash, net, and drop were more frequently performed by singles players in the last stroke of the rallies. However, stroke effectiveness and the effects of stroking actions on the point outcome failed to be quantified. Our findings may provide important insights for coaches to improve singles players’ stroking performances in the last stroke of the rallies. Moreover, no significant effects were found for the importance of rally on the winners and errors of both the men’s and women’s singles players, Gómez-Ruano et al. (2020c) argued that the context-related variables can affect the variability of match performance, and the critical periods (e.g., game point) could be considered a match-context constraint that impact the players’ scoring performance. However, this is not the case in the current study; the probabilities of achieving winners and errors did not correspondingly increase and decrease for either the men’s or women’s singles players during the key rally. In addition, great differences were found in the number of influencing factors between the men’s errors and women’s errors; only the defensive actions had a significant effect on the errors of the men’s singles players, while only the importance of rally showed a non-significant effect on the errors of the women’s singles players. The results indicate that the women’s singles players’ performances in the last strokes were more sensitive than men’s singles players to the technical variables and the match contexts. This finding may partly support the conclusion from Abian-Vicen et al. (2013), who found that women’s singles players obtained more unforced errors than men’s singles players during match play.

This study identified the effects of the technical variables and match contexts of the last stroke in rallies on the point outcome for both men’s and women’s singles players in elite badminton matches. The findings of the current study may provide some practical applications for coaches and players in the development of specific training interventions and the decision-making process during match play. We recommend that offensive actions, such as smash, can be used more frequently during rallies, and that a conservative playing strategy, especially at the end of the rallies, may not help the players to score points. This is also in line with the trend that badminton matches are more intense and competitiveness is increasing between players (Abián et al., 2014). As longer rallies may help them to achieve a better point outcome, women’s singles players could be advised to play the match more patiently. In addition, the women’s singles players were more easily affected by the match contexts than the men’s singles players. Therefore, they need to prepare specific match strategies for different match situations.

Some limitations of the present research should be noted and addressed in further studies. Firstly, as the performances of the last strokes are affected by previous actions during the rallies, analysis without the consideration of the entire stroking sequences of the rallies would lead to the loss of some key information about the stroking patterns. Secondly, the opponents’ match behaviors have impacts on the performance of the last strokes, and the stroking actions of the opponents also need to be recorded in order to analyze the dynamic interactions between players. Thirdly, the temporal variables should be included in future research to describe the temporal structure of the rallies, which may provide important insights for the understanding of the effects of physical and cognitive fatigue on the point outcome.

## Conclusion

In conclusion, the current study identified the key influencing factors of the point outcome from the perspective of the last stroke of the rallies in the elite badminton championships. In general, the point outcome of women’s singles players may more easily be affected by the technical and context-related variables than those of men’s singles players, especially the context-related variables. Specifically, the forehand and overhead strokes were negatively associated with the probability of achieving the winners, and defensive actions presented the greatest effects on the winners and errors of both men’s and women’s singles players. The selected technical and context-related variables had limited impacts on the errors of the men’s singles players, while they had great impacts on the errors of the women’s singles players. The match contexts had positive effects on the winners and negative effects on the errors of both sexes. These findings may help coaches and players to understand the scoring patterns of men’s and women’s singles players based on the performance of the last strokes of the rallies, leading to the development of specific training drills and match tactics and strategies.

## Data Availability Statement

The original contributions presented in the study are included in the article/supplementary material, further inquiries can be directed to the corresponding author.

## Author Contributions

YS, QY, and PC: conceptualization. YS and QY: methodology. YS: formal analysis, data collection, and writing—original draft preparation. QY: software and visualization. QY, M-ÁG-R, and PC: writing—review and editing. M-ÁG-R and PC: supervision. YS, M-ÁG-R, and PC: funding acquisition. Project administration: School of Physical Education and Sport Training, Shanghai University of Sport. All authors have read and agreed to the published version of the manuscript.

## Conflict of Interest

The authors declare that the research was conducted in the absence of any commercial or financial relationships that could be construed as a potential conflict of interest.

## Publisher’s Note

All claims expressed in this article are solely those of the authors and do not necessarily represent those of their affiliated organizations, or those of the publisher, the editors and the reviewers. Any product that may be evaluated in this article, or claim that may be made by its manufacturer, is not guaranteed or endorsed by the publisher.

## Footnotes

1
http://tv.cctv.com/cctv5
